# Glutamic-Pyruvic Transaminase 1 Facilitates Alternative Fuels for Hepatocellular Carcinoma Growth—A Small Molecule Inhibitor, Berberine

**DOI:** 10.3390/cancers12071854

**Published:** 2020-07-09

**Authors:** Wei Guo, Hor-Yue Tan, Sha Li, Ning Wang, Yibin Feng

**Affiliations:** School of Chinese Medicine, The University of Hong Kong, Hong Kong, China; guoweixt@connect.hku.hk (W.G.); hyhtan@hku.hk (H.-Y.T.); lishaha@hku.hk (S.L.); ckwang@hku.hk (N.W.)

**Keywords:** metabolic reprogramming, hepatocellular carcinoma, glucose–alanine cycle, GPT1, berberine

## Abstract

Metabolic reprogramming is an essential hallmark of cancer. Besides the “Warburg effect”, cancer cells also actively reprogram amino acid metabolism to satisfy high nutritional demands in a nutrient-poor environment. In the glucose–alanine cycle, exogenous alanine taken up by hepatocytes is converted to pyruvate via glutamic-pyruvic transaminases (GPTs). However, the precise role of the glucose–alanine cycle in hepatocellular carcinoma (HCC) remains elusive. The current study revealed that alanine, as an alternative energy source, induced the metabolic reprogramming of HCC cells via activation of the downstream glucose–alanine cycle and thus promoted HCC growth in nutrient-depleted conditions. Further overexpression and loss-of-function studies indicated that GPT1 was an essential regulator for alanine-supplemented HCC growth. Combining molecular docking and metabolomics analyses, our study further identified a naturally occurring alkaloid, berberine (BBR), as the GPT1 inhibitor in HCC. Mechanically, BBR-mediated metabolic reprogramming of alanine-supplemented HCC via GPT1 suppression attenuated adenosine triphosphate (ATP) production and thus suppressed HCC growth. In conclusion, our study suggests that GPT1-mediated alanine–glucose conversion may be a potential molecular target for HCC therapy. Further demonstration of BBR-mediated metabolic reprogramming of HCC would contribute to the development of this Chinese medicine-derived compound as an adjuvant therapy for HCC.

## 1. Introduction

Hepatocellular carcinoma (HCC) is the most common primary hepatic malignancy with complex risk factors, and ranks third in worldwide causes of cancer-related deaths [1,2]. Its prognosis is poor, as its mortality rate is similar to its incidence. There are three main conventional therapeutics for HCC: surgical resection, chemotherapy, and radiotherapy. Surgical resection is suitable for only a small number of HCC patients due to poor hepatic conditions at a late diagnosis. As the main therapies for HCC, chemotherapy and radiotherapy present serious side effects, such as hematological toxicity and liver and kidney dysfunction [3]. Moreover, the increasing acquired resistance reduces the therapeutic efficacy of these two main therapies [4]. Therefore, the development of more effective and less toxic therapeutic drugs is urgently needed.

Cell metabolism has a pivotal role in cancer, and metabolic reprogramming is one of the hallmarks of cancer [5]. Compared with normal cells, cancer cells show a distinct metabolic phenotype, which promotes their rapid proliferation and growth [6]. For example, even in aerobic conditions, cancer cells acquire energy with the shift from the highly efficient oxidative phosphorylation pathway to less efficient glycolysis, which is famously known as the “Warburg effect” [7]. In addition to glycolysis, glutaminolysis catabolizes glutamine to meet the biosynthetic and bioenergetic requirements of cancer cells [8]. Interestingly, except for the two primary nutrients, glucose and glutamine [9], cancer cells are also able to acquire alternative energy sources from a nutrient-poor environment to sustain their rapid proliferation [10]. Gluconeogenesis is central for glucose homeostasis through the use of non-carbohydrate carbon substrates to generate pyruvate and then fuel energy [11]. As a main gluconeogenic precursor, alanine, together with glycerol, glutamine, and lactate, accounts for over 90% of total gluconeogenesis [12]. Alanine could be used in gluconeogenesis to produce cellular adenosine triphosphate (ATP) and macromolecule intermediates [13]. It was recently reported that autophagy-dependent alanine secretion by stroma-associated pancreatic stellate cells (PSCs) fueled the tricarboxylic acid (TCA) cycle of pancreatic cancer cells [14]. However, the potential role of alanine as an alternative resource for HCC cells has been largely unexplored.

As an intermediary enzyme that reversibly catalyzes the transamination from α-ketoglutarate and alanine to generate glutamate and pyruvate, glutamic-pyruvic transaminase (GPT), also named alanine transaminase (ALT), plays an essential role in amino acid metabolism and gluconeogenesis [11]. The important role of this specific enzyme GPT in various kinds of cancers has been highlighted in several studies [15,16,17,18,19,20]. However, the clinical significance of GPT in HCC has been less discussed. There are two GPT isoforms, GPT1 and GPT2, in mammalians [21]. GPT2 has been widely suggested to be a critical determinant for the tumorigenesis of different cancers. For example, in colorectal cancer (CRC), phosphatidylinositol-4,5-bisphosphate 3-kinase catalytic subunit alpha (PIK3CA) mutations upregulate mitochondrial GPT2 to reprogram glutamine metabolism, which facilitates the rapid proliferation of CRC cells by converting more glutamine to a-ketoglutarate to replenish the TCA cycle and produce ATP [17]. Depletion of GPT2 in colon cancer cells attenuates their colony growth [20]. However, few studies have been performed on the regulation of GPT1 in cancer. Encoded by different genes, these two isoforms, GPT1 and GPT2, possess similar enzymatic activity but have different Km and Kcat values and different cellular and tissue distribution. Basically, as a cytoplasmic protein, GPT1 predominantly appears in the liver, intestines, and kidneys, while, as a mitochondrial protein, GPT2 is primarily expressed in the pancreas, heart, and brain [22]. Whether GPT1 also possesses a cancer-promoting function, like GPT2, in regulating energy metabolism and supporting cancer cell growth is yet to be determined.

The goal of this study was to investigate the role of the glucose–alanine cycle in mediating HCC growth in nutrient-depleted conditions, with a focus on GPT1 expression in HCC cells, and to reveal a potent inhibitor of GPT1 for the treatment of HCC.

## 2. Results

### 2.1. Alanine as an Alternative Energy Source Supports HCC Growth in a Nutrient-Poor Environment

**Figure 1:**
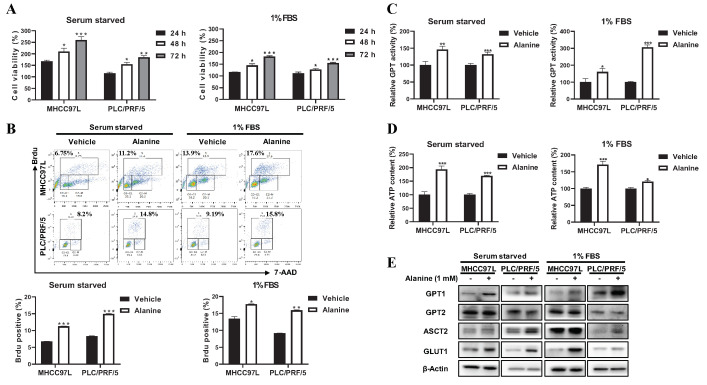
Alanine as an alternative energy source activated its downstream glucose–alanine cycle to promote HCC (hepatocellular carcinoma) growth in nutrient-depleted conditions. (**A**) The cell viability analysis of MHCC97L and PLC/PRF/5 cells under nutrient-deprived or low-nutrient-culture conditions following alanine supplementation for 24, 48, and 72 h. (**B**) The BrdU incorporation analysis of MHCC97L and PLC/PRF/5 cells under nutrient-deprived or low-nutrient-culture conditions following alanine supplementation for 24 h. (**C**) The GPT (glutamic-pyruvic transaminase) activity levels of MHCC97L and PLC/PRF/5 cells under nutrient-deprived or low-nutrient-culture conditions following alanine supplementation for 24 h. (**D**) The ATP (adenosine triphosphate) content levels of MHCC97L and PLC/PRF/5 cells under nutrient-deprived or low-nutrient-culture conditions following alanine supplementation for 24 h. (**E**) The representative Western blot panels of GPT1, GPT2, ASCT2 (alanine, serine, and cysteine-preferring transporter 2), and GLUT1 (glucose transporter 1) protein levels in MHCC97L and PLC/PRF/5 cells under nutrient-deprived or low-nutrient-culture conditions following alanine supplementation for 24 h. All experiments were conducted in triplicate unless otherwise stated. * *P* < 0.05, ** *P* < 0.01, *** *P* < 0.001.

A general characteristic of cancer cells that allows them to outcompete their neighbors is the ability to acquire necessary nutrients in a frequently nutrient-scarce microenvironment [6]. Apart from the commonly known nutrients, glucose and glutamine, cancer cells are also able to exploit diverse nutrient sources from a nutrient-poor environment to sustain their rapid proliferation. Alanine, together with the gluconeogenic precursor glycerol, glutamine, and lactate, accounts for over 90% of total gluconeogenesis [12]. We therefore hypothesize that a nonessential amino acid, alanine, is crucial for supporting HCC growth in nutrient-depleted conditions. To test this hypothesis, we supplemented alanine into nutrient-deprived or low-nutrient-cultured HCC cells. It was observed that the exogenous supplementation of alanine significantly enhanced HCC cells’ growth in nutrient-deprived or low-nutrient-culture conditions, which occurred in a time-dependent manner (Figure 1A). Consistent with the results from the HCC cell viability test, alanine supply substantially promoted HCC cell proliferation, as observed from the increased BrdU incorporated cell percentage (Figure 1B). The results indicated the indispensable role of alanine in supporting nutrient-deprived or low-nutrient-cultured HCC cell growth. GPT, also named ALT, is a critical enzyme that is responsible for the reversible conversion of alanine to pyruvate [11]. Observing enhanced HCC growth under alanine-rich conditions, we examined whether exogenous alanine supplementation increased GPT activity levels in nutrient-deprived or low-nutrient-cultured HCC cells. As expected, the addition of alanine significantly increased GPT activity levels under both nutrient-deprived and low-nutrient conditions (Figure 1C). Moreover, the alanine supply elevated the cellular ATP content levels, the metabolic endpoint in nutrient-deprived or low-nutrient-cultured HCC cells (Figure 1D). GPT is encoded by two isoenzymes, GPT1 and GPT2. In agreement with the GPT activity, the protein expression of GPT1 but not GPT2 was also increased following alanine supplementation (Figure 1E and Figure S1). Glucose transporter 1 (GLUT1) and the alanine, serine, and cysteine-preferring transporter 2 (ASCT2, also named SLC1A5) proteins are essential transporters to respectively transport glucose and alanine from the extracellular environment to the cell and are involved in the glucose–alanine cycle. Their expressions were similarly increased in response to exogenous alanine (Figure 1E and Figure S1). Altogether, these findings suggested that alanine, as an alternative energy source, activated its downstream glucose–alanine cycle to promote HCC growth in nutrient-depleted conditions.

### 2.2. Overexpression of GPT1 in the Activated Glucose–Alanine Cycle Promotes HCC Growth

In the glucose–alanine cycle, exogenous alanine taken up by hepatocytes is converted to pyruvate via transamination, which is mediated by GPTs [23]. GPT1 is localized in the cytoplasm, while GPT2 is mostly in the mitochondrial matrix [24]. Previous observation of a significant increase of GPT1 protein expression instead of GPT2 upon alanine supplementation led us to hypothesize that GPT1 activation is essential to sustain alternative energy for HCC growth under alanine-rich conditions. To confirm the role of GPT1 in HCC cells, we generated stable HCC cell lines by transfecting the GPT1 CRISPR activation plasmid into MHCC97L and PLC/PRF/5 cells. The overexpression of GPT1 increased the mRNA levels of GPT1 in MHCC97L and PLC/PRF/5 cells (Figure S2A). Consistently, the overexpression of GPT1 enhanced the protein level of GPT1 by 50% in MHCC97L and by 64% in PLC/PRF/5 cells (Figure S2B). Interestingly, under high-nutrient conditions (full medium: 25 mM glucose, 4 mM glutamine, 10% serum), although the overexpression of GPT1 significantly increased GPT enzymatic activity by 240% in MHCC97L and by 190% in PLC/PRF/5 cells (Figure S2C), there was no significant difference in the cell viability (Figure S2D) and apoptosis (Figure S2E) of MHCC97L and PLC/PRF/5 cells after the overexpression of GPT1, indicating a close correlation of GPT1 with nutrient-depleted conditions. To confirm the indispensable role of alanine as an alternative energy resource via GPT1-mediated alanine–glucose conversion in a nutrient-poor environment, the effects of exogenous alanine supplementation on the cellular GPT activity and ATP content levels of GPT1-overexpressed HCC cells were examined. It was observed that the alanine supply significantly elevated the cellular GPT activity and ATP content levels of GPT1-overexpressed HCC cells in a nutrient-poor environment (Figure S3A,B).

**Figure 2:**
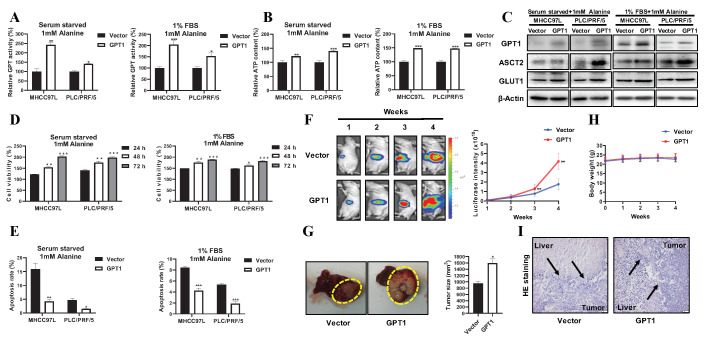
Overexpression of GPT1 in the activated glucose–alanine cycle promoted HCC growth. (**A**) The GPT activity levels of MHCC97L and PLC/PRF/5 cells with or without stable GPT1 overexpression under alanine-rich conditions for 24 h. (**B**) The ATP content levels of MHCC97L and PLC/PRF/5 cells with or without stable GPT1 overexpression under alanine-rich conditions for 24 h. (**C**) The representative Western blot panels of GPT1, ASCT2, and GLUT1 protein levels in MHCC97L and PLC/PRF/5 cells with or without stable GPT1 overexpression under alanine-rich conditions for 24 h. (**D**) The cell viability analysis of MHCC97L and PLC/PRF/5 cells with or without stable GPT1 overexpression under alanine-rich conditions for 24, 48, and 72 h. (**E**) The cell apoptosis analysis of MHCC97L and PLC/PRF/5 cells with or without stable GPT1 overexpression under alanine-rich conditions for 24 h. (**F**) The representative images and statistical graph of tumor growth with or without stable GPT1 overexpression based on luciferase signals during the experiment (*n* = 5). (**G**) The representative images and statistical graph of tumor size with or without stable GPT1 overexpression at the end of the experiment (*n* = 5). (**H**) The statistical graph of body weight of tumor-bearing mice with or without stable GPT1 overexpression during the experiment (*n* = 5). (**I**) The representative H&E staining images of the boundary of tumor and liver tissues with or without stable GPT1 overexpression at the end of the experiment (*n* = 5). All experiments were conducted in triplicate unless otherwise stated. * *P* < 0.05, ** *P* < 0.01, *** *P* < 0.001.

Thus, we next investigated the role of GPT1 in sustaining alanine as an alternative energy source for HCC under nutrient-poor conditions with alanine supplementation. Of note, the overexpression of GPT1 substantially increased the cellular GPT activity and ATP content levels in the HCC cells under alanine-rich conditions (Figure 2A,B). In accordance with GPT activity, the overexpression of GPT1 increased the protein expression of GPT1 (Figure 2C and Figure S4). Moreover, the expression levels of ASCT2 and GLUT1 were also enhanced by GPT1 overexpression (Figure 2C and Figure S4). These results indicated that GPT1 overexpression activated the glucose–alanine cycle of nutrient-deprived or low-nutrient-cultured HCC cells with alanine supplementation. More importantly, the overexpression of GPT1 promoted HCC cell growth under alanine-rich conditions in a time-dependent manner (Figure 2D). Consistently, the overexpression of GPT1 reduced the HCC cell apoptotic rate (Figure 2E and Figure S5A) as compared with control cells. All these results further indicated that GPT1 may be the promoting gene in potentiating alanine-mediated HCC growth and proliferation. To further examine the clinical relevance of GPT1 expression in HCC, we established an orthotopic HCC implantation mouse model by implanting subcutaneous grown MHCC97L cells, with or without stable GPT1 overexpression, on the mice liver lobes (Figure S5B). Consistent with the in vitro cell proliferation data, the overexpression of GPT1 in tumor cells exacerbated the orthotopic hepatic tumor growth after three weeks of implantation (Figure 2F). By the end of the four-week experiment, the tumor-bearing animals were sacrificed, and the hepatic tumors were removed. The mice implanted with GPT1-overexpressed HCC cells showed increased tumor size compared to the control mice implanted with wild-type paired HCC cells (Figure 2G). However, GPT1 overexpression had a minimal effect on the body weight of tumor-bearing mice (Figure 2H). Further histological analysis showed that GPT1 overexpression stimulated the orthotropic HCC cells to invade normal hepatic tissue, as evidenced by an irregular and invasive edge in the boundary of tumor and normal hepatic tissues (Figure 2I). Overall, GPT1 overexpression improved the glucose–alanine cycle, and thereby promoted HCC growth and progression.

### 2.3. Inhibition of GPT1 Expression Reverses Alanine-Mediated HCC Growth

**Figure 3:**
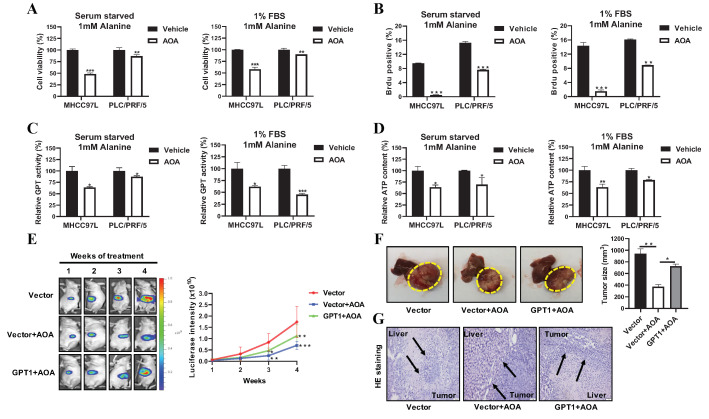
Inhibition of GPT1 with AOA (aminooxyacetate) potently suppressed alanine-mediated HCC growth. (**A**) The cell viability analysis of MHCC97L and PLC/PRF/5 cells under alanine-rich conditions following AOA treatment for 24 h. (**B**) The BrdU incorporation analysis of MHCC97L and PLC/PRF/5 cells under alanine-rich conditions following AOA treatment for 24 h. (**C**) The GPT activity levels of MHCC97L and PLC/PRF/5 cells under alanine-rich conditions following AOA treatment for 24 h. (**D**) The ATP content levels of MHCC97L and PLC/PRF/5 cells under alanine-rich conditions following AOA treatment for 24 h. (**E**) The representative images and statistical graph of tumor growth with or without stable GPT1 overexpression based on luciferase signals during the AOA treatment (*n* = 5). (**F**) The representative images and statistical graph of tumor size with or without stable GPT1 overexpression at the end of AOA treatment (*n* = 5). (**G**) The representative H&E staining images of the boundary of tumor and liver tissues with or without stable GPT1 overexpression at the end of AOA treatment (*n* = 5). All experiments were conducted in triplicate unless otherwise stated. * *P* < 0.05, ** *P* < 0.01, *** *P* < 0.001.

To confirm the role of GPT1 in HCC growth, we used a loss-of-function approach to combat the increased HCC-expressed GPT1. Reasoning that alanine supplementation resulted in GPT1 overexpression, we treated the alanine-supplemented HCC cells with aminooxyacetate (AOA), a general inhibitor of enzymatic activity of transaminase [19]. It was observed that the addition of AOA reduced HCC cell viability in the presence of alanine (Figure 3A). Consistently, AOA reversed the alanine-mediated HCC proliferation in nutrient-deprived conditions (Figure 3B and Figure S6A). These results suggested that GPT1 inhibition potently reversed alanine-mediated HCC growth and proliferation. AOA primarily suppresses alanine transaminase, so the incubation of HCC cells with AOA substantially reduced the GPT activity levels (Figure 3C) and cellular ATP content levels (Figure 3D). To further investigate the clinical relevance of GPT1 inhibition in HCC, AOA was given as treatment to orthotopic HCC-implanted mice with or without stable GPT1 overexpression. Of note, AOA caused low toxicity to the mice, as evidenced by a slightly decreased body weight (Figure S7A). A histological analysis of the main organs also revealed that AOA had low toxicity toward the heart and lungs of the mice (Figure S7B). As shown in Figure S6B, the orthotopic tumor GPT1 expression was significantly suppressed after a four-week treatment with AOA, which was remarkably rescued by the overexpression of GPT1. Administration of AOA to orthotopic HCC-implanted mice resulted in hepatic tumor shrinkage after three weeks of treatment, while overexpression of GPT1 decreased the inhibitory effects of AOA on tumor growth in mice (Figure 3E). By the end of the treatment, the dissected hepatic tumor from the AOA treatment mice group without GPT1 overexpression showed about a 60% tumor size reduction, while the dissected hepatic tumor from the AOA treatment mice group with GPT1 overexpression showed only a 20% tumor size reduction as compared to the untreated group of mice without GPT1 overexpression (Figure 3F). Further histological examination revealed that AOA-treated hepatic tumors had a non-invasive growth pattern and distinct liver and tumor borders, suggesting that AOA treatment potently reduced intra-tumoral growth, which was rescued by the overexpression of GPT1 (Figure 3G). Overall, these findings suggested that the inhibition of GPT1 by AOA potently suppressed alanine-mediated HCC growth and proliferation.

### 2.4. Targeting GPT1 by a Small Molecule Berberine Suppresses HCC Growth

**Figure 4:**
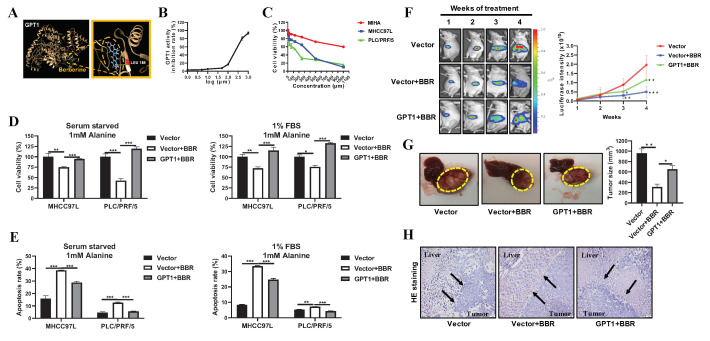
Targeting GPT1 by a small molecule BBR (berberine) suppressed HCC growth. (**A**) The in silico molecular docking analysis of the binding capacity of BBR with different binding sites of GPT1. (**B**) The GPT1 activity inhibition rate of increasing concentrations of BBR against the recombinant human GPT1 protein. (**C**) The cell viability of MHCC97L, PLC/PRF/5, and MIHA cells when exposed to increasing concentrations of BBR (7.8125–1000 µM) for 24 h in full medium. (**D**) The cell viability analysis of MHCC97L and PLC/PRF/5 cells with or without stable GPT1 overexpression under alanine-supplemented conditions after BBR treatment for 24 h. (**E**) The cell apoptosis analysis of MHCC97L and PLC/PRF/5 cells with or without stable GPT1 overexpression under alanine-supplemented conditions after BBR treatment for 24 h. (**F**) The representative images and statistical graph of tumor growth with or without stable GPT1 overexpression based on luciferase signals during the BBR treatment (*n* = 5). (**G**) The representative images and statistical graph of tumor size with or without stable GPT1 overexpression at the end of BBR treatment (*n* = 5). (**H**) The representative H&E staining images of the boundary of tumor and liver tissues with or without stable GPT1 overexpression at the end of BBR treatment (*n* = 5). All experiments were conducted in triplicate unless otherwise stated. * *P* < 0.05, ** *P* < 0.01, *** *P* < 0.001.

Given that GPT1 may be an oncogene in promoting HCC growth, we searched for a small molecule that specifically binds to and suppresses GPT1 activity. Molecular docking is a powerful tool to extract target-specific and active molecules from Chinese herbal medicine [25]. We performed an in silico molecular docking analysis of the binding capacity of chemical components (ligands) with different binding sites of GPT1 (targets). Interestingly, we found that a naturally occurring alkaloid, berberine (BBR), which is majorly present in the Chinese herb *Coptis chinensis*, showed the most potent binding potency with GPT1 protein. The 3D structure of the binding profile between BBR and GPT1 is shown in Figure 4A. In detail, BBR entered the substrate-binding region and bound to the active site of GPT1. The binding energy was −6.76 kcal/mol and the fill fitness was −4038.91 kcal/mol. In addition, it was observed that the enzymatic activity of the recombinant human GPT1 protein was inhibited by BBR in a dose-dependent manner (Figure 4B). Our previous studies revealed that BBR potently suppressed HCC growth and liver-to-lung metastasis [26,27]. When exposed to increasing concentrations of BBR (7.8125–1000 µM) for 24 h, a dose-dependent decrease in cell viability was observed in both HCC cells. The half-maximal inhibitory concentration (IC50) of BBR was 255.3 μM for MHCC97L cells and 103.5 μM for PLC/PRF/5 cells. However, when the normal human liver MIHA cells were treated with BBR (7.8125–1000 µM) for 24 h, the proliferation inhibition rates were lower than 40%, indicating a selective inhibitory role of BBR on HCC cells (Figure 4C). Low-toxicity doses of BBR for 24 h were chosen for the following experiments, namely 100 μM for MHCC97L cells and 50 μM for PLC/PRF/5 cells, respectively. Of note, the overexpression of GPT1 efficiently rescued HCC cells from BBR-induced cell death and apoptosis under alanine-supplemented conditions (Figure 4D,E and Figure S8A). These results indicated that BBR suppressed alanine-mediated HCC growth primarily through deactivating GPT1. To further underscore the role of GPT1 in mediating the inhibitory effects of BBR on HCC growth, we treated orthotopic HCC-implanted mice with or without GPT1 overexpression with BBR. Of note, no severe toxicity of BBR was observed in the mice, as evidenced by the comparable body weight and histological analysis of the main organs between the control and BBR-treated groups (Figure S9). As shown in Figure S8B, overexpression of GPT1 efficiently reversed the BBR-mediated inhibition of orthotopic tumor GPT1 expression. It was observed that hepatic tumors in the BBR-treated group of mice grew more slowly. Compared with the BBR-treated group of mice without GPT1 overexpression, the luciferase signals of orthotopic hepatic tumors were moderately enhanced in BBR-treated mice-bearing hepatic tumors with GPT1 overexpression (Figure 4F). At the end of treatment, the tumor size was also pronouncedly increased in BBR-treated hepatic tumors with GPT1 overexpression compared to BBR-treated wild-type tumors (Figure 4G). Furthermore, histological examination showed that the edges at the boundary of the tumor and liver became irregular and invasive in BBR-treated hepatic tumors with GPT1 overexpression (Figure 4H). All these findings suggested that the overexpression of GPT1 significantly neutralized the inhibitory effects of BBR on HCC cell and orthotopic tumor growth.

### 2.5. Berberine Mediates Metabolic Fluctuations Primarily by Regulating the Glucose–Alanine Cycle in HCC

**Figure 5:**
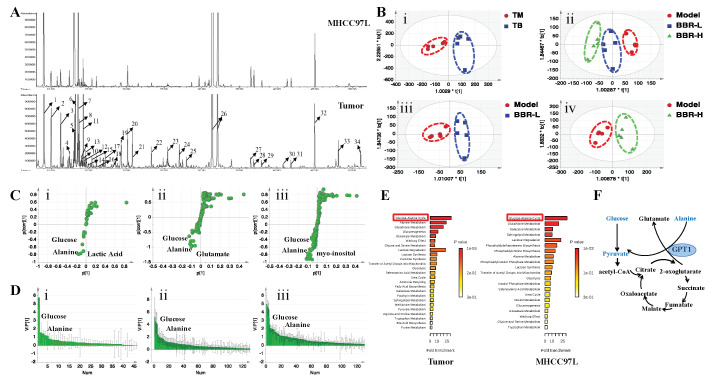
BBR mediated metabolic fluctuations primarily by regulating the glucose–alanine cycle in HCC. (**A**) The typical GC/MS (gas chromatography/mass spectrometry) TICs (total ion chromatograms) of tumor and cell samples. 1, Lactate; 2, Alanine; 3, 3-Hydroxybutyric acid; 4, Valine; 5, Urea; 6, Ethanolamine; 7, Glycerol; 8, Phosphonic acid; 9, Isoleucine; 10, Proline; 11, Glycine; 12, Butanedioic acid; 13, Pentadecane; 14, Uracil; 15, Serine; 16, Threonine; 17, beta-Alanine; 18, Succinic acid; 19, Malic acid; 20, 5-Oxoproline; 21, Hydroxyproline; 22, Glutamate; 23, Tetracosane; 24, Phosphorylethanolamine; 25, Citric acid; 26, Glucose; 27, Talose; 28, Palmitic Acid; 29, Myo-Inositol; 30, Oleic acid; 31, Stearic acid; 32, Tetracosane (IS); 33, 1-Monopalmitin; and 34, Glycerol monostearate. (**B**) The OPLS-DA (orthogonal partial least squares-discriminant analysis) score plots based on the data derived from the tumor and MHCC97L samples after BBR treatment (*n* = 5). (i) Tumor samples: Model vs. BBR; (ii) MHCC97L samples: Model vs. BBR-L (50 μM) vs. BBR-H (100 μM); (iii) MHCC97L samples: Model vs. BBR-L (50 μM); and (iv) MHCC97L samples: Model vs. BBR-H (100 μM). The S-loading plots (**C**) and VIP (variable importance in the projection) score plots (**D**) based on the data derived from the tumor and MHCC97L samples after BBR treatment. (i) Tumor samples: Model vs. BBR; (ii) MHCC97L samples: Model vs. BBR-L (50 μM); and (iii) MHCC97L samples: Model vs. BBR-H (100 μM). (**E**) Metabolic pathway analyses of tumor and MHCC97L samples based on the variable metabolites of significant difference after BBR treatment. (**F**) Schematic illustration of the metabolic flux involved in the glucose–alanine cycle.

In order to further evaluate the metabolic reprogramming effects of BBR on HCC and explore the potential targeted pathways, metabolic profiling analysis of both mice hepatic tumor and MHCC97L cell samples with or without BBR treatment was conducted by gas chromatography/mass spectrometry (GC/MS)-based metabolomics and multivariate statistical analysis. The typical GC/MS total ion chromatograms (TICs) of the tumor and cell are shown in Figure 5A. The endogenous metabolites were identified by comparing the retention times and mass spectra characteristics or searching the mass spectral database library NIST 2005. In total, 53 metabolites from tumors and 25 metabolites from MHCC97L cells were identified, including sugars, fatty acids, amino acids, and organic acids. The orthogonal partial least squares-discriminant analysis (OPLS-DA) score plots based on the metabolites of tumor and MHCC97L cell samples showed that BBR-treated groups were clearly separated from the model group, indicating a different metabolic pattern among these groups (Figure 5B). Of note, there was a dose-dependent response of MHCC97L cells to BBR treatment. Those ions farthest away from the main cluster in the S-loading plots indicated the most influential ions, which were responsible for the separation between the model and BBR-treated groups (Figure 5C). Moreover, those ions with a variable importance in the projection (VIP) value greater than 1.0 in the VIP score plots also indicated the most influential ions (Figure 5D). In detail, compared with the model group, glucose, alanine, glutamate, lactate, and myo-inositol levels were decreased, while the levels of valine, glycine, serine, threonine, and 5-oxoproline were increased in the BBR-treated groups. Specifically, the metabolic pathway analysis of the most variable metabolites from mice hepatic tumor and MHCC97L cell samples further indicated that BBR may mediate metabolic fluctuations primarily via regulating the glucose–alanine cycle in HCC both in vitro and in vivo (Figure 5E). The schematic illustration of the metabolic flux involved in the glucose–alanine cycle is shown in Figure 5F. From the above results, we identified that BBR mediated metabolic fluctuations primarily by regulating the glucose–alanine cycle in HCC from a metabolic perspective.

### 2.6. Berberine-Suppressed GPT1 Reverses the Dysregulated Energy Homeostasis in HCC

**Figure 6:**
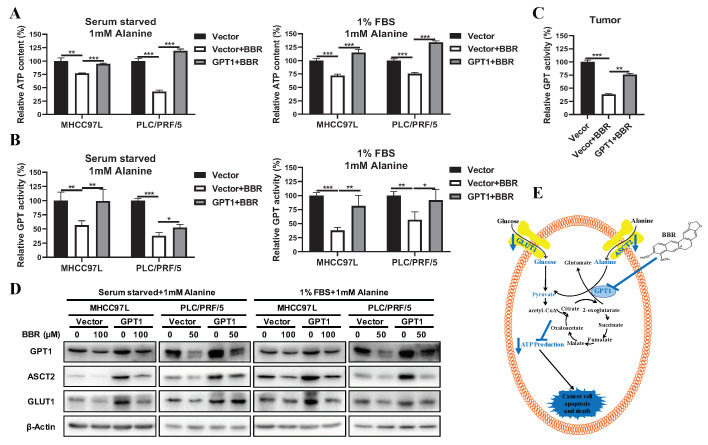
BBR-suppressed GPT1 reversed the dysregulated energy homeostasis in HCC. (**A**) The ATP content levels of MHCC97L and PLC/PRF/5 cells with or without stable GPT1 overexpression under alanine-supplemented conditions after BBR treatment for 24 h. (**B**) The GPT activity levels of MHCC97L and PLC/PRF/5 cells with or without stable GPT1 overexpression under alanine-supplemented conditions after BBR treatment for 24 h. (**C**) The GPT activity levels of the tumor with or without stable GPT1 overexpression at the end of BBR treatment (*n* = 5). (**D**) The representative Western blot panels of GPT1, ASCT2, and GLUT1 protein levels of MHCC97L and PLC/PRF/5 cells with or without stable GPT1 overexpression under alanine-supplemented conditions after BBR treatment for 24 h. (**E**) Schematic illustration of the regulatory mechanism underlying the inhibitory effects of BBR on HCC. All experiments were conducted in triplicate unless otherwise stated. * *P* < 0.05, ** *P* < 0.01, *** *P* < 0.001.

BBR is a potent glucose-reducing agent [28], yet whether BBR regulates energy homeostasis in HCC cells remains unclear. To determine whether BBR-mediated GPT1 regulated the energy metabolism of alanine-supplemented HCC cells, ATP content levels were detected in alanine-supplemented HCC cells with or without GPT1 overexpression following BBR treatment. It was observed that BBR treatment significantly decreased alanine-supplemented HCC cellular ATP content levels, while the overexpression of GPT1 reversed the decreased ATP content levels induced by BBR (Figure 6A). Interestingly, BBR treatment significantly reduced GPT activity levels in alanine-supplemented HCC cells, and the effect was abolished by GPT1 overexpression (Figure 6B). Consistent with the in vitro observations, BBR treatment significantly reduced GPT activity levels in orthotopic tumor tissue, while this effect was reversed in BBR-treated HCC tumor tissue with GPT1 overexpression (Figure 6C). In accordance with the GPT activity, BBR treatment markedly decreased GPT1 expression level, while the overexpression of GPT1 significantly reversed the effect (Figure 6D and Figure S10). It was also observed that BBR reduced the expression levels of glucose–alanine cycle related proteins ASCT2 and GLUT1, while this effect was reversed in BBR-treated HCC cells with GPT1 overexpression under alanine-rich conditions (Figure 6D and Figure S10). In summary, these findings suggested that BBR-suppressed GPT1 expression reversed the alternatively activated glucose–alanine cycle in HCC cells, and thereby inhibited HCC growth.

## 3. Discussion

During excessive proliferation and growth, solid tumors including HCC are exposed to multiple stresses such as energy depletion, low nutrient availability, oxidative stress, and so on [29]. Tumors must alter the way they capture and use nutrients to satisfy high nutritional demands in a nutrient-poor environment [10]. In addition to the “Warburg effect”, cancer cells also actively modify their amino acid metabolism [10,30]. Amino acids could be the intermediate metabolites fueling various biosynthetic pathways, which are regulated by various transaminases via triggering amino acid-mediated TCA anaplerosis [16]. Recently, amino acid depletion therapies have become new therapeutics for cancer treatment. For example, leucine deprivation induced widespread apoptotic death of melanoma cells [30]. Alanine was suggested to feed tumor cells and promote tumorigenesis under nutrition-poor conditions [14,31,32]. In detail, the autophagy-dependent alanine secretion by stroma-associated PSCs could be utilized by pancreatic cancer cells [14]. The supplied alanine underwent transamination to generate pyruvate and then provided pancreatic cancer cells with energy. Such metabolic reprogramming of pancreatic cancer cells could free up glucose to be more available for other biosynthetic processes and facilitate tumor growth, especially in a nutrient-deprived environment. 

In our study, we found that the exogenous addition of alanine, as an alternative energy resource, could induce the metabolic reprogramming of HCC cells via activating the downstream glucose–alanine cycle and thus promote HCC growth in nutrient-depleted conditions. The shift to alanine as a fuel source reduced the tumor’s dependence on glucose, glutamine, and serum-derived nutrients, supplying the nutrients necessary for the rapid growth of HCC. GPTs, the alanine transaminases for the conversion from alanine to glucose, play an obligate role in amino acid metabolism and gluconeogenesis [11,33]. Further overexpression and loss-of-function studies indicated that the expression of GPT1, rather than GPT2, was responsible for alanine-supplemented HCC growth. It was observed that, in high-nutrient conditions, there was no significant difference in cell viability and apoptosis, although the overexpression of GPT1 significantly increased GPT enzymatic activity, indicating the close correlation of GPT1 with nutrient-depleted conditions. The indispensable role of alanine as an alternative energy source via GPT1-mediated alanine–glucose conversion in a nutrient-poor environment was evidenced by the elevated cellular GPT activity and ATP content levels of GPT1-overexpressed HCC cells in a nutrient-poor environment after alanine supply. A further overexpression study revealed that the overexpression of GPT1 led to expression of ASCT2 and GLUT1, suggesting that GPT1 in the upstream position is an essential molecule leading to the HCC cell’s metabolic switch to use alanine as an energy source during nutrient deprivation. AOA is a general inhibitor of enzymatic activity of transaminases, including GPTs. It has been reported that AOA is closely related to amino acid metabolism and shows pronounced antitumor effects in various preclinical investigations as a single agent [19]. Of note, the inhibition of GPT1 with AOA potently suppressed alanine-mediated HCC growth and proliferation.

BBR is a naturally occurring alkaloid and a principal component of the Chinese herb *Coptis chinensis*, which has been clinically used for the treatment of inflammation and cancer for centuries. Recently, its potential anti-HCC effects have also been suggested [26,27,34]. In detail, cytotoxic doses of BBR inhibited the proliferation [35] and induced the mitochondrial apoptosis and autophagic cell death of HCC cells [36]. Low-toxic doses of BBR inhibited the angiogenesis [26] and invasion of HCC cells [37]. Moreover, BBR inhibited in vivo tumor growth and the liver-to-lung metastasis of HCC cells [27]. Mechanically, BBR mediated transcriptional [27], post-transcriptional [35], and epigenetic [38] regulation of the oncogenic and tumor suppressor genes. In the current study, we investigated its anti-HCC property from the metabolic perspective. We found that BBR showed most potent binding potency with GPT1 protein based on an in silico molecular docking analysis. Of note, a direct inhibitory effect of BBR on GPT1 enzymatic activity was indicated, as evidenced by a dose-dependent inhibition of BBR on the enzymatic activity of the recombinant human GPT1 protein in vitro. A GC/MS-based metabolomics analysis of both mice hepatic tumor and HCC cells was conducted to further evaluate the metabolic reprogramming effects of BBR on HCC and explore the potential targeted pathways. The results of the metabolomics analysis revealed that the BBR-treated group showed a metabolic pattern significantly distinct from that of the model group. Of note, the metabolic pathway analysis of the most variable metabolites indicated that BBR mediated the metabolic reprogramming of HCC primarily by regulating the glucose–alanine cycle.

It was seen that the overexpression of GPT1 efficiently rescued HCC cells from BBR-induced cell death and apoptosis under alanine-supplemented conditions. Consistent with the in vitro observations, the in vivo experiments also indicated that BBR exhibited inhibitory effects on orthotopic tumor growth, which was significantly neutralized by the overexpression of GPT1. Interestingly, compared with the current transaminase inhibitor AOA, BBR showed no severe toxicity to normal cells and mice. Of note, BBR decreased the cellular GPT activity and ATP content levels, while the overexpression of GPT1 significantly reversed the effects. Mechanically, in our study, BBR reduced the expression levels of the glucose–alanine cycle-involved proteins GPT1, GLUT1, and ASCT2, which were obviously neutralized by GPT1 overexpression. As a rate-limiting transporter for glucose uptake, GLUT1 is enhanced in a subset of HCC patients. The role of BBR in decelerating the glucose metabolism of cancer cells via inhibiting GLUT1 has been suggested [39]. ASCT2 is essential to transport alanine from the extracellular environment to the cell and is positively associated with tumorigenesis [40]. The endpoint of BBR-mediated metabolic reprogramming of alanine-supplemented HCC via GPT1 suppression blocked ATP production and thus resulted in induced apoptosis and suppressed HCC growth. In general, this is the first comprehensive study to report that the GPT1 is closely associated with the regulation role of BBR in metabolic reprogramming of HCC by alanine–glucose conversion, which may be developed as a potential new molecular target for the treatment of HCC.

## 4. Materials and Methods

### 4.1. Reagents and Antibodies

Berberine chloride (PHR1502), AOA (#C13408), and alanine (A4349) were purchased from Sigma-Aldrich (St. Louis, MO, USA). Antibodies against GPT1 (ab231715) and GPT2 (ab80947) were purchased from Abcam (Cambridge, MA, USA). Antibodies against ASCT2 (5345S), GLUT1 (#12939) and β-actin (#4970) were bought from Cell Signaling Technology (Danvers, MA, USA).

### 4.2. Cell Line and Cell Culture

PLC/PRF/5 cells were bought from American Type Culture Collection (ATCC, Rockville, MD, USA). MHCC97L cells tagged with a luciferase reporter gene and normal human liver MIHA cells were kindly gifted by Professor Man Kwan from the Department of Surgery, the University of Hong Kong. For high-nutrient condition, cells were cultured in DMEM with high concentrations of glutamine and glucose (4 mM glutamine and 25 mM glucose) and 10% FBS and 1% penicillin/streptomycin. For nutrition-poor conditions, cells were cultured in the base DMEM (no glutamine and 25 mM glucose) without FBS or with 1% FBS and 1% penicillin/streptomycin. For both conditions, cells were incubated in a humidified chamber with 5% CO_2_ at 37 °C.

### 4.3. Plasmid and Transfection

GPT CRISPR activation plasmid was purchased from Santa Cruz Biotechnology (sc-402909-ACT, Santa Cruz, CA, USA). Both MHCC97L and PLC/PRF/5 cells were transfected with GPT1 CRISPR activation plasmid using FuGENE HD transfection reagent (Promega, Madison, USA) based on the manufacturer’s instructions. In brief, plasmid was mixed with FuGENE HD transfection reagent in the serum free DMEM. After incubation at room temperature for 15 min, cells were transfected with the mixture for another 48 h. Stable clones of GPT1-overexpressing MHCC97L and PLC/PRF/5 cells were established by long-term puromycin selection.

### 4.4. Cell Viability Assay

The cell viability of MIHA, MHCC97L, and PLC/PRF/5 cells exposed to BBR for 24 h under high-nutrient conditions was measured via MTT (3-(4,5-Dimethylthiazol-2-yl)-2,5-diphenyltetrazolium bromide) assay. In detail, 5000 cells/well were seeded in 96-well plates and incubated overnight. Then, cells were treated with increasing concentrations of BBR (0, 7.8125, 15.625, 31.25, 62.5, 125, 250, 500, and 1000 μM) for 24 h. For alanine supplement experiments under nutrition-poor conditions, 5000 cells/well were seeded in 96-well plates and incubated overnight. Then cells were treated with or without 1 mM alanine under nutrition-poor conditions for 24, 48, and 72 h. For GPT1 overexpression experiments under alanine-rich conditions, 5000 cells/well with or without stable GPT1 overexpression were seeded in 96-well plates and incubated overnight. Then the full medium was changed to the nutrition-poor medium with alanine supplementation and cells were further cultured for 24, 48, and 72 h. For AOA or BBR experiments under alanine-rich conditions, 5000 cells/well with or without stable GPT1 overexpression were seeded in 96-well plates and incubated overnight. Then the full medium was changed to the nutrition-poor medium with alanine supplementation and cells were treated with or without AOA (500 μM for both HCC cells) or BBR (100 μM for MHCC97L cells and 50 μM for PLC/PRF/5 cells) for 24 h. Afterwards, 10 μL of MTT (5 mg/mL) was added, followed by 4 h incubation. The supernatants were discarded and 100 μL of DMSO was added. The absorbance was tested at 595 nm by a microplate reader (Biorad, Hercules, CA, USA).

### 4.5. BrdU Incorporation Assay

A BrdU incorporation assay was performed by FITC BrdU Flow Kit (559619, BD Biosciences, San Jose, CA, USA) according to the manufacturer’s instructions to evaluate the cell proliferation. In detail, 24-h-treated cells were incubated with 10 μM BrdU (BD Pharmingen, San Jose, CA, USA) for 2 h at 37 °C, 5% CO_2_. Cells were then trypsinized and fixed, followed by permeabilization. Then, 30 μg of DNase was added to expose incorporated BrdU for 1 h. Staining was conducted with the FITC-tagged anti-BrdU antibody for 20 min at room temperature. The analysis was conducted on a Canto II flow cytometer (BD Biosciences, San Jose, CA, USA) within 1 h.

### 4.6. Annexin V/7-AAD Staining and Flow Cytometry

Annexin V/7-AAD staining was conducted by a PE Annexin V Apoptosis Detection Kit (559763, BD Biosciences, San Jose, CA, USA) according to the manufacturer’s instructions to evaluate the cell apoptosis. In detail, 24-h-treated cells were collected and stained with PE-conjugated Annexin V and 7-AAD for 15 min at room temperature in the dark. The analysis was conducted on a Canto II flow cytometer (BD Biosciences, San Jose, CA, USA) within 1 h.

### 4.7. ATP Content Assay

An ATP Colorimetric/Fluorometric Assay Kit (K354-100, Biovision, Milpitas, CA, USA) was applied to measure intracellular ATP content according to the manufacturer’s instructions. In detail, 24-h-treated cells were lysed with ATP assay buffer. A 10 kDa spin column was used for deproteinization. Then, 50 μL samples were mixed with 50 μL reaction mix (44 μL ATP assay buffer, 2 μL ATP probe, 2 μL ATP converter, and 2 μL developer) and incubated at room temperature for 30 min in the dark. The absorbance was tested at 570 nm by a microplate reader (Labsystems, Vantaa, Finland).

### 4.8. Orthotopic HCC Implantation Murine Model

All animal experimental procedures were approved by the Committee on the Use of Live Animals in Teaching and Research (CULATR), The University of Hong Kong (Document No.: 3776-15). In detail, the left waist of 5-week-old male BALB/c athymic nude mice was subcutaneously injected with 10 × 10^6^ luciferase-tagged MHCC97L cells with or without stable GPT1 overexpression (vector or GPT1). The subcutaneous tumor was collected and cut into small cubes (approximately 1 mm^3^) when it reached 1 cm in diameter. To establish the orthotopic HCC implantation murine model, the left liver lobes of 5-week-old male BALB/c athymic nude mice were orthotopically implanted with the small tumor cubes from subcutaneously grown MHCC97L cells with or without stable GPT1 overexpression (vector or GPT1). After one week, the successful tumor was examined with an in vivo live imaging system (Perkin-Elmer, Waltham, MA, USA) by intraperitoneally injecting luciferin (15 mg/kg). The mice bearing a tumor from wild-type paired MHCC97L cells (vector) were then randomly separated into model, AOA-, and BBR-treated groups receiving intraperitoneal injections of equivalent PBS, AOA (10 mg/kg/2 days), or BBR (10 mg/kg/2 days) respectively, for four consecutive weeks (*n* = 5 for each group). For mice bearing a tumor from GPT1-overexpressed MHCC97L cells (GPT1), three groups, namely model, AOA-, and BBR-treated groups, were created and treated as the mice bearing a tumor from wild-type paired MHCC97L cells (*n* = 5 for each group). The tumor growth was examined weekly by an in vivo live imaging system. Tissues were collected from the six groups of orthotopic HCC implantation mice.

### 4.9. Histology Examination

Collected tissues were fixed with 4% formalin buffer. Then, the paraffin-embedded blocks were prepared and cut into sections of 4 μm in thickness. Sections were stained with hematoxylin and eosin for histological examination.

### 4.10. GC/MS-Based Metabolomics Analysis

For tumor samples (*n* = 5 for each group), about 25 mg of tumor from the model and BBR-treated groups of mice with wild-type paired MHCC97L cells (vector, receiving equivalent PBS or 10 mg/kg/2 days BBR for four consecutive weeks) was added to 500 µL of 80% methanol and homogenized. For cell samples (*n* = 5 for each group), wild-type paired MHCC97L cells were treated with different concentrations of BBR (0, 50, and 100 μM) in full medium for 24 h. A total of 500 µL of 80% methanol was added after discarding the medium and freeze–melt cycles were conducted three times (freezing at −80 °C for 60 min and melting at 37 °C for 20 min). All samples were centrifuged at 12,000× *g* for 10 min at 4 °C. Following this step, 350 µL of the supernatant was evaporated to dryness in a vacuum. All dried samples were derivatized with 30 µL methoxylamine hydrochloride (15 mg/mL in pyridine) at 70 °C for 60 min, followed by 50 µL N-methyl-N (trimethylsilyl) trifluoroacetamide (MSTFA) with 1% trimethylchlorosilane (TMCS) at 40 °C for 30 min. Then, 700 µL of heptane (0.1 mg/mL of tetracosane as internal standard) was added to the solution. Finally, after centrifugation at 3000× *g* for 10 min, 600 µL of the supernatant was collected to GC vial.

Samples were acquired using a GC-(EI)-Q-MS (6890N-GC with 5973-MS ECD/NPD, Agilent, Santa Clara, CA, USA). Briefly, a 1-µL sample was injected into a 30 m × 0.25 mm DB-5 MS capillary column using a splitless injection. Helium was used as a carrier gas with a constant flow of 1.0 mL/min. The initial temperature of the GC oven was held at 60 °C for 3 min, followed by an increase to 140 °C at a rate of 7 °C/min for 4 min. Then, the column temperature was increased to 180 °C at a rate of 5 °C/min for 6 min. Finally, the temperature was increased to 280 °C at a rate of 5 °C/min for another 4 min. After a solvent delay of 8 min, MS detection was employed with electron ionization mode at 70 eV ionization energy and full scan mode in a range of 50–650 m/z. Chemstation software was used to convert all the GC/MS raw files to NetCDF format. The XCMS toolbox (http://metlin.scripps.edu/download/) was applied to process data. Multivariate analyses were conducted by SIMCA-P+ 13.0 software.

### 4.11. Determination of Enzymatic Activity Inhibition

To verify the direct inhibitory effect of BBR on GPT1, the enzymatic activity of purified recombinant human GPT1 protein (ab206804, Abcam, Cambridge, MA, USA), with or without BBR, was measured by an ALT/SGPT Liqui-UV Test kit (2930-500, Stanbio, Boerne, TX, USA) according to the manufacturer’s instructions. In detail, 500 mM L-Alanine, 15 mM α-KG, 1200 units/L lactate dehydrogenase, 0.18 mM NADH, and 0.01 mg/mL recombinant human GPT1 protein were combined with increasing concentrations of BBR (0, 1, 5, 10, 50, 100, 500, and 1000 μM) in a 200-μL reaction. The absorbance rate at 340 nm was recorded by a microplate reader (Labsystems, Vantaa, Finland) for about 5 min.

### 4.12. Intracellular ALT Activity Assay

The ALT activity in both cells and tissue was measured by an ALT/SGPT Liqui-UV Test kit (2930-500, Stanbio, Boerne, TX, USA) according to the manufacturer’s instructions. In detail, cells (1 × 10^6^) or tissue (50 mg) were rapidly lysed or homogenized with 200 μL ALT buffer (R1) and centrifuged at 12,000× *g* for 10 min at 4 °C. ALT working reagent was prepared in a ratio of 5 parts of buffer (R1) to 1 part of enzyme (R2). For each sample and blank control well of a 96-well UV plate, 200 μL working reagent was added and warmed to 37 °C. Then, 20 μL of cell or tissue homogenate was added to the sample well and gently mixed. The absorbance rate at 340 nm was recorded by a microplate reader (Labsystems, Vantaa, Finland) for about 5 min.

### 4.13. Real-Time PCR

Total RNA was extracted with the Trizol method (Takara, Kyoto, Japan) as the template for first-strand synthesis. Gene expression was measured by SYBR Green assay (Takara, Kyoto, Japan) on the LC480 platform (Roche, Nutley, NJ, USA). The sequences of the primers for particular genes were as follows: GGGTTCGCAGTTCCACTCATT (forward) and CCGCACACTCATCAGCTTCA (reverse) for GPT1; and CTCTGAGGCTCTTTTCCAGCC (forward) and TAGAGGTCTTTACGGATGTCAACGT (reverse) for β-actin.

### 4.14. Immunoblotting

Total protein was extracted with RIPA buffer with proteinase and phosphatase inhibitors. Proteins were separated via sodium dodecyl sulfate-polyacrylamide gel electrophoresis (SDS-PAGE) and transferred to a PVDF membrane (Roche, Nutley, NJ, USA). Then, the membrane was blocked with 5% BSA for 2 h at room temperature and incubated with primary antibodies at 4 °C overnight, followed by incubation with secondary antibodies for 2 h at room temperature. The blot was measured with a chemiluminescence imaging system (Biorad, Hercules, CA, USA) using an ECL kit (GE Healthcare, Chicago, IL, USA).

### 4.15. Statistical Analysis

Prism 6 software was used for the statistical analysis. All experiments were performed in triplicates unless otherwise stated, and data are shown as mean ± SD. Differences between the two groups were analyzed using a two-tailed Student’s t-test, and a one-way ANOVA was used for multiple comparisons (groups of more than two). *P* < 0.05 was considered to be statistically significant.

## 5. Conclusions

In our study, we demonstrated that alanine, as an alternative energy source, activated its downstream glucose–alanine cycle, which is crucial for HCC growth under nutrient-deprived conditions. Further investigation revealed that the expression of GPT1 was responsible for the activation of the glucose–alanine cycle for HCC growth. Moreover, inhibition of GPT1 reversed alanine-supplemented HCC growth. Combining molecular docking and metabolomics analyses, our study further identified a naturally occurring alkaloid, BBR, as the GPT1 inhibitor for HCC growth. Mechanically, BBR-mediated metabolic reprogramming of alanine-supplemented HCC via GPT1 inhibition blocked ATP production and thus suppressed HCC growth (Figure 6E). In conclusion, our current study indicates that GPT1-mediated alanine–glucose conversion may be a potential molecular target for HCC therapy. Further demonstration of BBR-mediated metabolic reprogramming of HCC would contribute to the development of this Chinese medicine-derived compound as an adjuvant therapy for HCC.

## Supplementary Materials

The following are available online at https://www.mdpi.com/2072-6694/12/7/1854/s1, Figure S1: The quantitation results of Figure 1E. Figure S2: The stably overexpressed GPT1 in MHCC97L and PLC/PRF/5 cells was conformed in mRNA (A), protein (B) and activity (C) levels. The overexpression of GPT1 showed no effects on cell viability (D) and apoptosis (E) of HCC cells under high-nutrient condition. Figure S3: Alanine supply significantly elevated the cellular GPT activity (A) and ATP content (B) levels of GPT1 overexpressed HCC cells under nutrient-poor environment. Figure S4: The quantitation results of Figure 2C. Figure S5: The cell apoptosis analysis (A) of MHCC97L and PLC/PRF/5 cells with or without stable GPT1 overexpression under alanine-rich conditions for 24 h and the GPT1 protein levels (B) in the tumor with or without stable GPT1 overexpression. Figure S6: The BrdU incorporation analysis (A) of MHCC97L and PLC/PRF/5 cells under alanine-rich conditions following AOA treatment for 24 h and the GPT1 protein levels (B) in the tumor with or without stable GPT1 overexpression at the end of AOA treatment. Figure S7: The statistical graph of body weight (A) and the representative H&E staining images of the main organs (B) between the control and AOA-treated groups. Figure S8: The cell apoptosis analysis (A) of MHCC97L and PLC/PRF/5 cells under alanine-rich conditions following BBR treatment for 24 h and the GPT1 protein levels (B) in the tumor with or without stable GPT1 overexpression at the end of BBR treatment. Figure S9: The statistical graph of body weight (A) and the representative H&E staining images of the main organs (B) between the control and BBR-treated groups. Figure S10: The quantitation results of Figure 6D. Figure S11: Whole blot showing all the bands with molecular weight markers on the Western.

## References

[1] Schafer D.F., Sorrell M.F. (1999). Hepatocellular carcinoma. Lancet.

[2] Best J., Schotten C., Theysohn J.M., Wetter A., Müller S., Radünz S., Schulze M., Canbay A., Dechêne A., Gerken G. (2016). Novel implications in the treatment of hepatocellular carcinoma. Ann. Gastroenterol..

[3] Wang Z., Qi F., Cui Y., Zhao L., Sun X., Tang W., Cai P. (2018). An update on Chinese herbal medicines as adjuvant treatment of anticancer therapeutics. Biosci. Trends.

[4] Lou J., Yao P., Tsim K.W., Tism K.W.K. (2019). Cancer Treatment by Using Traditional Chinese Medicine: Probing Active Compounds in Anti-multidrug Resistance during Drug Therapy. Curr. Med. Chem..

[5] Ward P.S., Thompson C.B. (2012). Metabolic Reprogramming: A Cancer Hallmark Even Warburg Did Not Anticipate. Cancer Cell.

[6] Pavlova N., Thompson C.B. (2016). The Emerging Hallmarks of Cancer Metabolism. Cell Metab..

[7] Liberti M., Locasale J.W. (2016). The Warburg Effect: How Does it Benefit Cancer Cells?. Trends Biochem. Sci..

[8] Jin L., Alesi G.N., Kang S. (2015). Glutaminolysis as a target for cancer therapy. Oncogene.

[9] Heiden M.G.V., Cantley L.C., Thompson C.B. (2009). Understanding the Warburg Effect: The Metabolic Requirements of Cell Proliferation. Science.

[10] Kamphorst J.J., Nofal M., Commisso C., Hackett S.R., Lu W., Grabocka E., Heiden M.G.V., Miller G., Drebin J.A., Bar-Sagi D. (2015). Human pancreatic cancer tumors are nutrient poor and tumor cells actively scavenge extracellular protein. Cancer Res..

[11] Qian K., Zhong S., Xie K., Yu D., Yang R., Gong D.-W. (2015). Hepatic ALT isoenzymes are elevated in gluconeogenic conditions including diabetes and suppressed by insulin at the protein level. Diabetes/Metab. Res. Rev..

[12] Gerich J.E. (1993). Control of glycaemia. Baillieres Clin. Endocrinol. Metab..

[13] Schindhelm R., Diamant M., Dekker J.M., Tushuizen M.E., Teerlink T., Heine R.J. (2006). Alanine aminotransferase as a marker of non-alcoholic fatty liver disease in relation to type 2 diabetes mellitus and cardiovascular disease. Diabetes/Metab. Res. Rev..

[14] Sousa C.M., Biancur D.E., Wang X., Halbrook C.J., Sherman M.H., Zhang L., Kremer D., Hwang R.F., Witkiewicz A.K., Ying H. (2016). Pancreatic stellate cells support tumour metabolism through autophagic alanine secretion. Nature.

[15] Cao Y., Lin S.-H., Wang Y., Chin Y.E., Kang L., Mi J. (2017). Glutamic Pyruvate Transaminase GPT2 Promotes Tumorigenesis of Breast Cancer Cells by Activating Sonic Hedgehog Signaling. Theranostics.

[16] Smith B., Schafer X.L., Ambeskovic A., Spencer C.M., Land H., Munger J. (2016). Addiction to Coupling of the Warburg Effect with Glutamine Catabolism in Cancer Cells. Cell Rep..

[17] Hao Y., Samuels Y., Li Q., Krokowski D., Guan B.-J., Wang C., Jin Z., Dong B., Cao B., Feng X. (2016). Oncogenic PIK3CA mutations reprogram glutamine metabolism in colorectal cancer. Nat. Commun..

[18] Coloff J.L., Murphy J.P., Braun C.R., Harris I., Shelton L.M., Kami K., Gygi S.P., Selfors L.M., Brugge J.S. (2016). Differential Glutamate Metabolism in Proliferating and Quiescent Mammary Epithelial Cells. Cell Metab..

[19] Korangath P., Teo W.W., Sadik H., Han L., Mori N., Huijts C.M., Wildes F., Bharti S., Zhang Z., Santa-Maria C.A. (2015). Targeting Glutamine Metabolism in Breast Cancer with Aminooxyacetate. Clin. Cancer Res..

[20] Weinberg F., Hamanaka R., Wheaton W.W.E., Weinberg S., Joseph J., Lopez M., Kalyanaraman B., Mutlu G.M., Budinger G.R.S., Chandel N.S. (2010). Mitochondrial metabolism and ROS generation are essential for Kras-mediated tumorigenicity. Proc. Natl. Acad. Sci. USA.

[21] Glinghammar B., Rafter I., Lindström A.-K., Hedberg J.J., Andersson H.B., Lindblom P., Berg A.-L., Cotgreave I. (2009). Detection of the mitochondrial and catalytically active alanine aminotransferase in human tissues and plasma. Int. J. Mol. Med..

[22] Yang R.-Z., Park S., Reagan W.J., Goldstein R., Zhong S., Lawton M., Rajamohan F., Qian K., Liu L., Gong D.-W. (2008). Alanine aminotransferase isoenzymes: Molecular cloning and quantitative analysis of tissue expression in rats and serum elevation in liver toxicity. Hepatology.

[23] Felig P. (1973). The glucose-alanine cycle. Metabolism.

[24] Ye J., Gu Y., Zhang F., Zhao Y., Yuan Y., Hao Z., Sheng Y., Li W.Y., Wakeham A., Cairns R.A. (2016). IDH1 deficiency attenuates gluconeogenesis in mouse liver by impairing amino acid utilization. Proc. Natl. Acad. Sci USA.

[25] Muhammad S.A., Fatima N. (2015). In silico analysis and molecular docking studies of potential angiotensin-converting enzyme inhibitor using quercetin glycosides. Pharmacogn. Mag..

[26] Tan H.Y., Wang N., Tsao S.-W., Zhang Z., Feng Y. (2013). Suppression of Vascular Endothelial Growth Factor via Inactivation of Eukaryotic Elongation Factor 2 by Alkaloids in Coptidis rhizome in Hepatocellular Carcinoma. Integr. Cancer Ther..

[27] Tsang C.M., Cheung K.C., Cheung Y.C., Man K., Lui V.W.Y., Tsao S.W., Feng Y. (2015). Berberine suppresses Id-1 expression and inhibits the growth and development of lung metastases in hepatocellular carcinoma. Biochim. Biophys. Acta (BBA)—Mol. Basis Dis..

[28] Pirillo A., Catapano A.L. (2015). Berberine, a plant alkaloid with lipid- and glucose-lowering properties: From in vitro evidence to clinical studies. Atherosclerosis.

[29] Kroemer G., Pouysségur J. (2008). Tumor Cell Metabolism: Cancer’s Achilles’ Heel. Cancer Cell.

[30] Sheen J.-H., Zoncu R., Kim D., Sabatini D.M. (2011). Defective Regulation of Autophagy upon Leucine Deprivation Reveals a Targetable Liability of Human Melanoma Cells In Vitro and In Vivo. Cancer Cell.

[31] Kamphorst J.J., Gottlieb E. (2016). Cancer metabolism: Friendly neighbours feed tumour cells. Nature.

[32] Dickson I. (2016). Pancreatic cancer: Stromal-cancer cell crosstalk supports tumour metabolism. Nat. Rev. Gastroentero. Hepato..

[33] Ouyang Q., Nakayama T., Baytaş O., Davidson S.M., Yang C., Schmidt M., Lizarraga S.B., Mishra S., Ei-Quessny M., Niaz S. (2016). Mutations in mitochondrial enzyme GPT2 cause metabolic dysfunction and neurological disease with developmental and progressive features. Proc. Natl. Acad. Sci. USA.

[34] Wang M.-D., Wu H., Fu G.-B., Zhang H.-L., Zhou X., Tang L., Dong L.-W., Qin C.-J., Huang S., Zhao L.-H. (2016). Acetyl-coenzyme A carboxylase alpha promotion of glucose-mediated fatty acid synthesis enhances survival of hepatocellular carcinoma in mice and patients. Hepatology.

[35] Wang N., Wang X., Tan H.-Y., Li S., Tsang C.M., Tsao S.-W., Feng Y. (2016). Berberine Suppresses Cyclin D1 Expression through Proteasomal Degradation in Human Hepatoma Cells. Int. J. Mol. Sci..

[36] Wang N., Feng Y., Zhu M., Tsang C.-M., Man K., Tong Y., Tsao S.-W. (2010). Berberine induces autophagic cell death and mitochondrial apoptosis in liver cancer cells: The cellular mechanism. J. Cell. Biochem..

[37] Wang N., Feng Y., Lau E.P.W., Tsang C., Ching Y.P., Man K., Tong Y., Nagamatsu T., Su W., Tsao S. (2010). F-Actin Reorganization and Inactivation of Rho Signaling Pathway Involved in the Inhibitory Effect of Coptidis Rhizoma on Hepatoma Cell Migration. Integr. Cancer Ther..

[38] Wang N., Zhu M., Wang X., Tan H.-Y., Tsao S.-W., Feng Y. (2014). Berberine-induced tumor suppressor p53 up-regulation gets involved in the regulatory network of MIR-23a in hepatocellular carcinoma. Biochim. Biophys. Acta (BBA)—Bioenerg..

[39] Mao L., Chen Q., Gong K., Xu X., Xie Y., Zhang W., Cao H., Hu T., Hong X., Zhan Y.-Y. (2018). Berberine decelerates glucose metabolism via suppression of mTOR-dependent HIF-1α protein synthesis in colon cancer cells. Oncol. Rep..

[40] Van Geldermalsen M. (2016). ASCT2/SLC1A5 controls glutamine uptake and tumour growth in triple-negative basal-like breast cancer. Oncogene.

