# Supplementary material for: Glutamic-Pyruvic Transaminase 1 Facilitates Alternative Fuels for Hepatocellular Carcinoma Growth—A Small Molecule Inhibitor, Berberine
Source: Cancers (Basel). 2020 Jul 9;12(7):1854. doi: 10.3390/cancers12071854 (PMC7408817; doi:10.3390/cancers12071854)
Supplement: Supplementary file 1 [file cancers-12-01854-s001.pdf]

Supplementary Materials: Glutamic-pyruvic Transaminase 1 Facilitates Alternative Fuels for Hepatocellular Carcinoma Growth—A Small Molecule Inhibitor, Berberine

Wei Guo, Hor-Yue Tan, Sha Li, Ning Wang, and Yibin Feng*

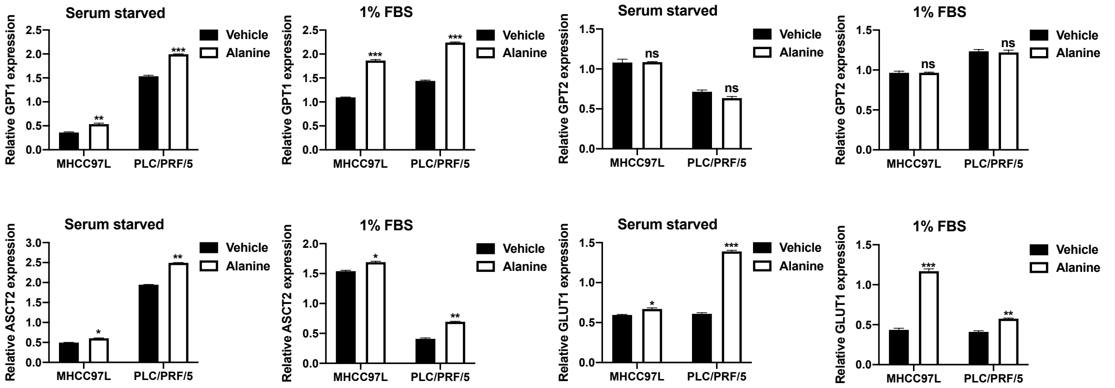

Figure S1. The quantitation results of Figure 1E.

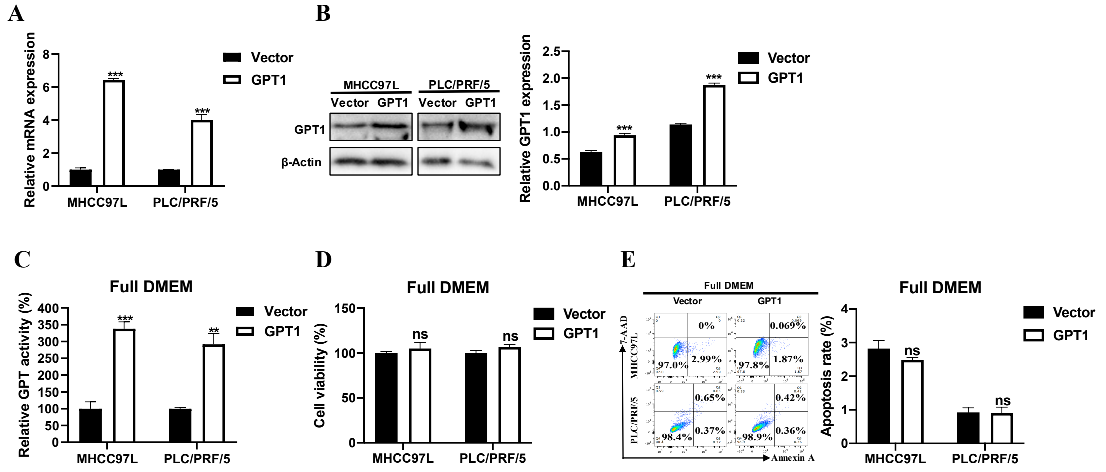

Figure S2. The stably overexpressed GPT1 in MHCC97L and PLC/PRF/5 cells was conformed in mRNA (A), protein (B) and activity (C) levels. The overexpression of GPT1 showed no effects on cell viability (D) and apoptosis (E) of HCC cells under high-nutrient condition.

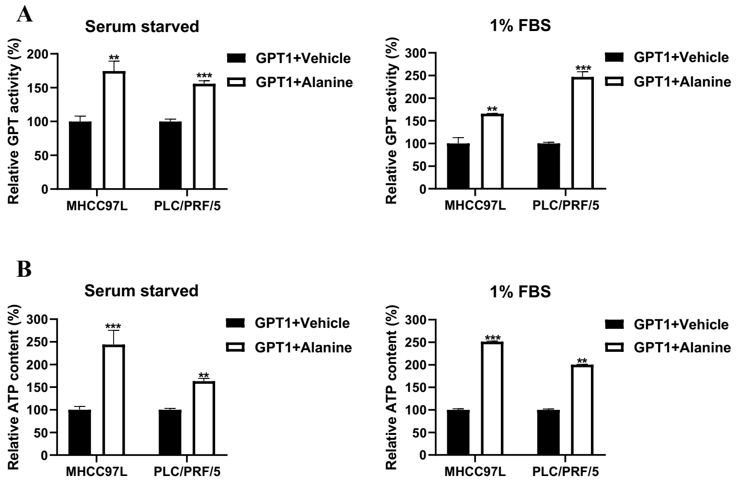

Figure S3. Alanine supply significantly elevated the cellular GPT activity (A) and ATP content (B) levels of GPT1 overexpressed HCC cells under nutrient-poor environment.

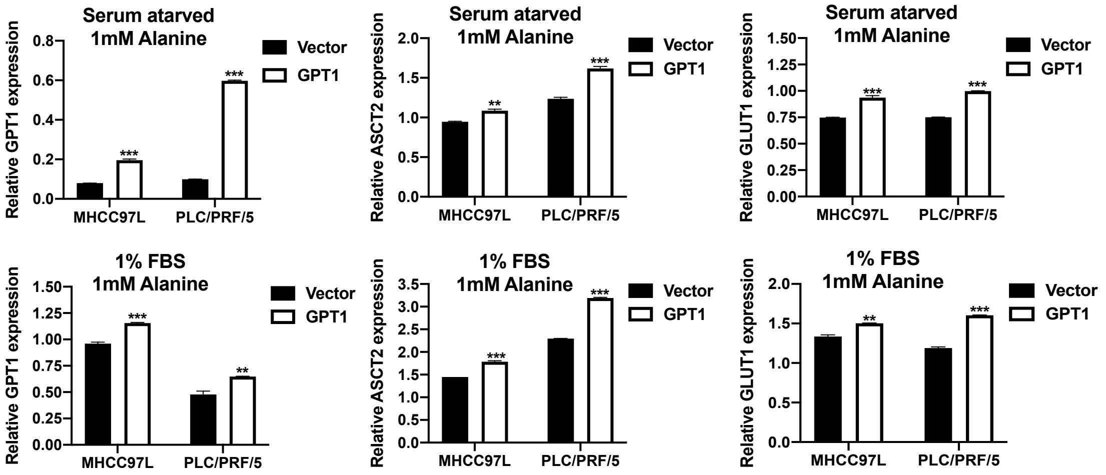

Figure S4. The quantitation results of Figure 2C.

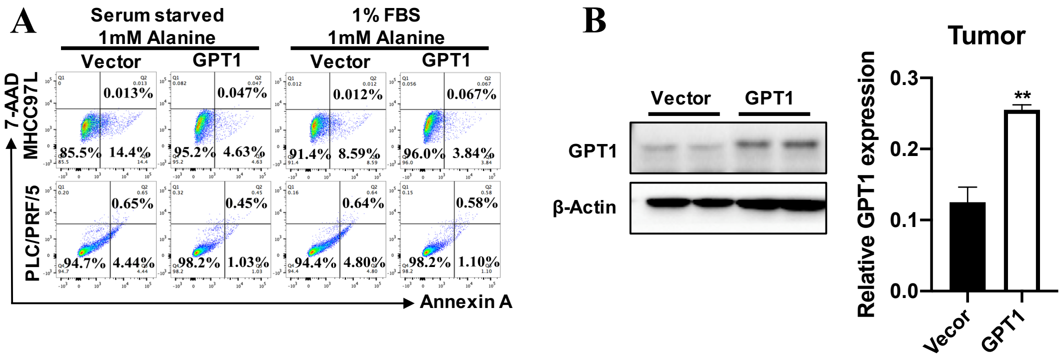

Figure S5. The cell apoptosis analysis (A) of MHCC97L and PLC/PRF/5 cells with or without stable GPT1 overexpression under alanine-rich conditions for 24 h and the GPT1 protein levels (B) in the tumor with or without stable GPT1 overexpression.

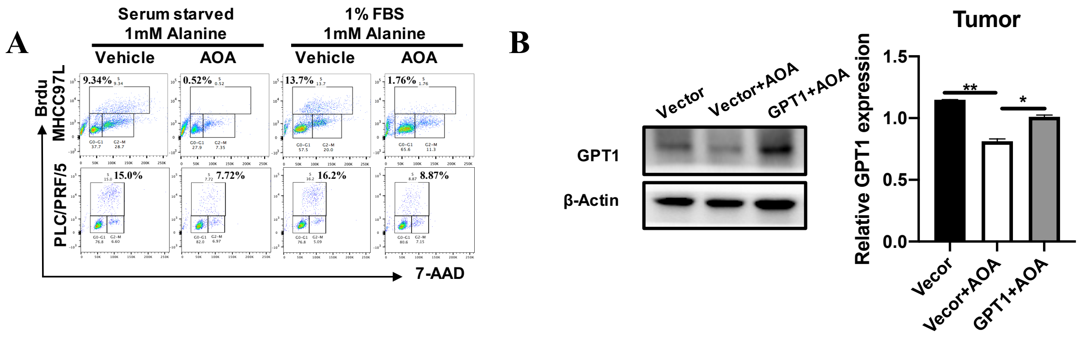

Figure S6. The BrdU incorporation analysis (A) of MHCC97L and PLC/PRF/5 cells under alanine-rich conditions following AOA treatment for 24 h and the GPT1 protein levels (B) in the tumor with or without stable GPT1 overexpression at the end of AOA treatment.

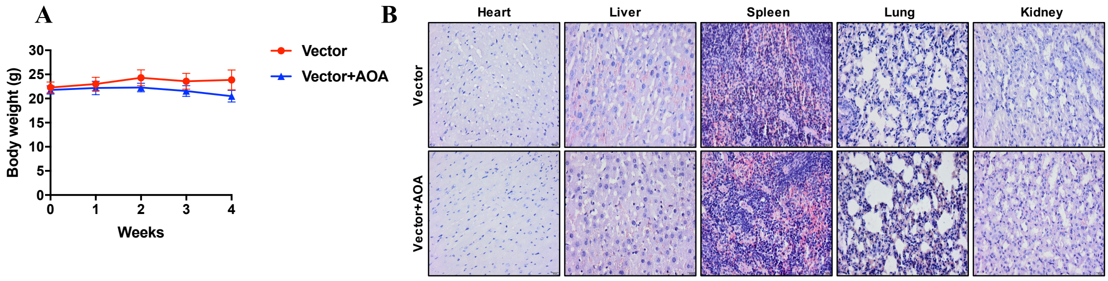

Figure S7: The statistical graph of body weight (A) and the representative H&E staining images of the main organs (B) between the control and AOA-treated groups.

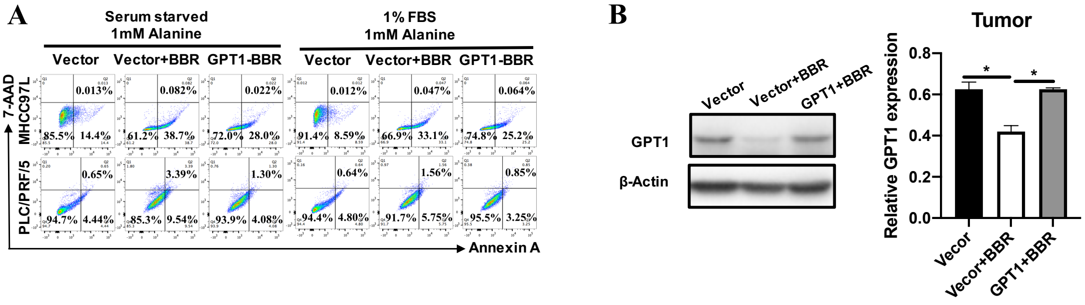

Figure S8. The cell apoptosis analysis (A) of MHCC97L and PLC/PRF/5 cells under alanine-rich conditions following BBR treatment for 24 h and the GPT1 protein levels (B) in the tumor with or without stable GPT1 overexpression at the end of BBR treatment.

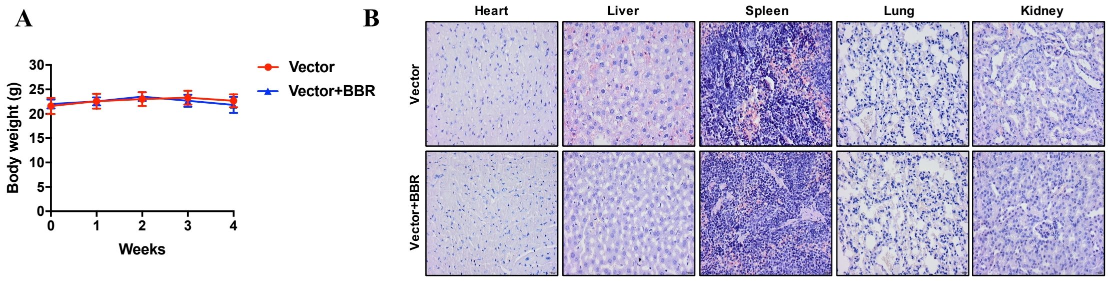

Figure S9. The statistical graph of body weight (A) and the representative H&E staining images of the main organs (B) between the control and BBR-treated groups.

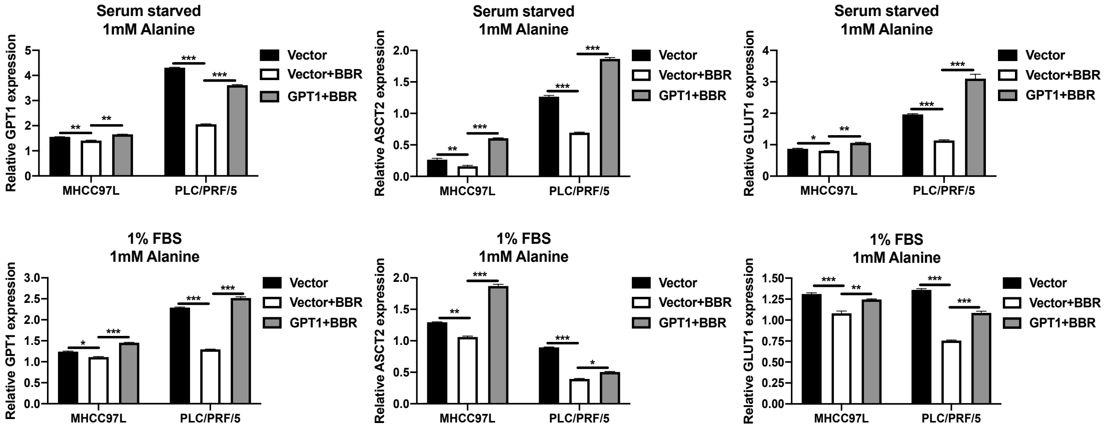

Figure S10. The quantitation results of Figure 6D.

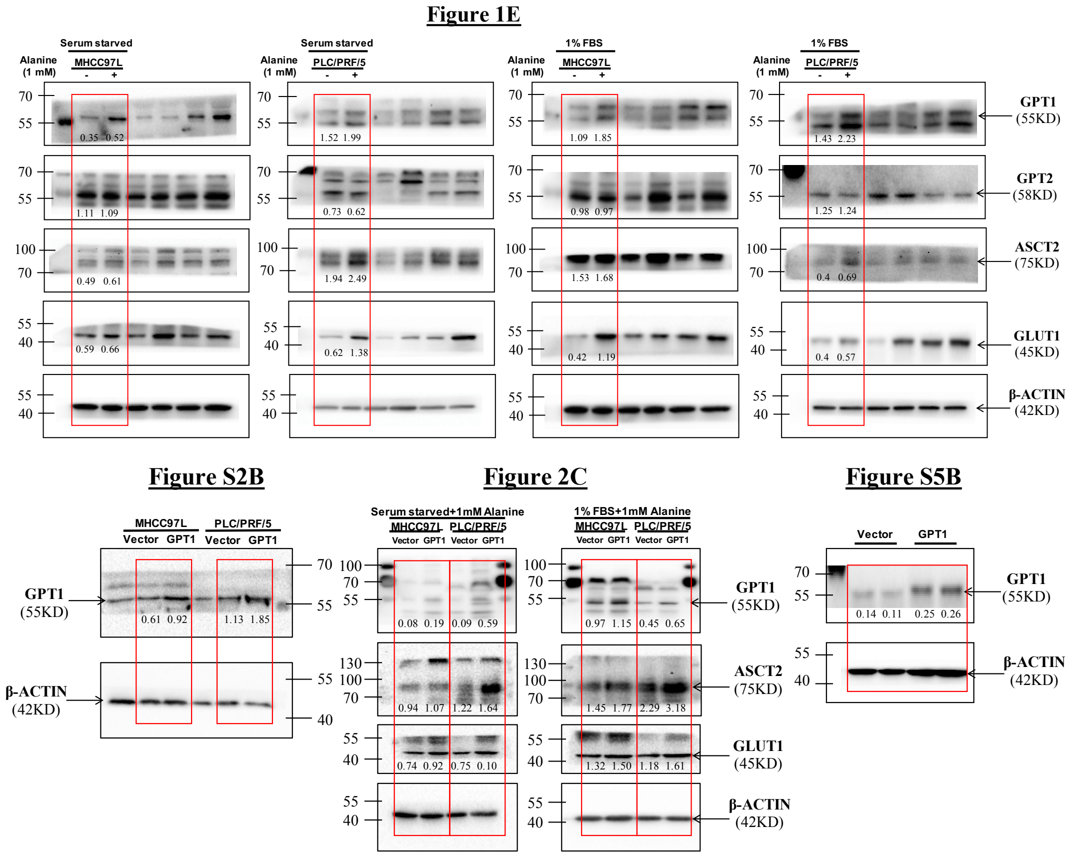

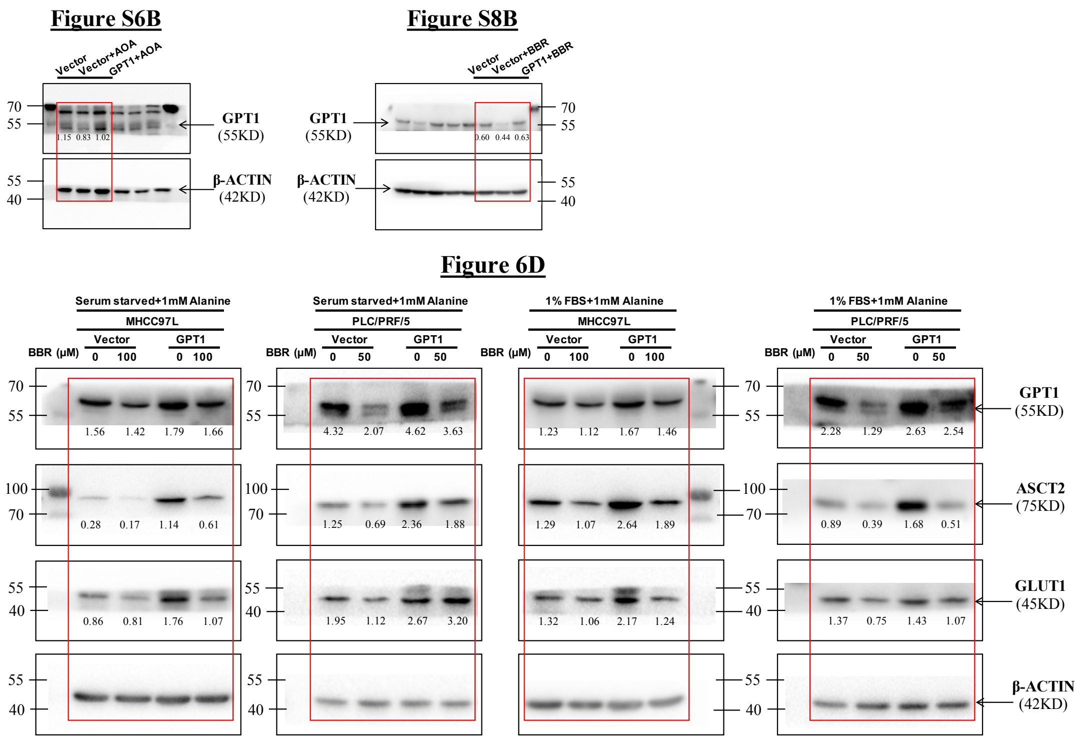

Figure S11. Whole blot showing all the bands with molecular weight markers on the Western.
